# The History and Horizons of Microscale Neural Interfaces

**DOI:** 10.3390/mi9090445

**Published:** 2018-09-06

**Authors:** Takashi D. Y. Kozai

**Affiliations:** 1 Department of Bioengineering, University of Pittsburgh, Pittsburgh, PA 15261, USA; tdk18@pitt.edu; 2 Center for the Neural Basis of Cognition, University of Pittsburgh, Pittsburgh, PA 15213, USA; 3 Center for Neuroscience, University of Pittsburgh, Pittsburgh, PA 15261, USA; 4 McGowan Institute of Regenerative Medicine, University of Pittsburgh, Pittsburgh, PA 15212, USA; 5 NeuroTech Center, University of Pittsburgh Brain Institute, Pittsburgh, PA 15260, USA

**Keywords:** micromachine, neuroscience, biocompatibility, training, education, diversity, bias, BRAIN Initiative, multi-disciplinary, micro-electromechanical systems (MEMS)

## Abstract

Microscale neural technologies interface with the nervous system to record and stimulate brain tissue with high spatial and temporal resolution. These devices are being developed to understand the mechanisms that govern brain function, plasticity and cognitive learning, treat neurological diseases, or monitor and restore functions over the lifetime of the patient. Despite decades of use in basic research over days to months, and the growing prevalence of neuromodulation therapies, in many cases the lack of knowledge regarding the fundamental mechanisms driving activation has dramatically limited our ability to interpret data or fine-tune design parameters to improve long-term performance. While advances in materials, microfabrication techniques, packaging, and understanding of the nervous system has enabled tremendous innovation in the field of neural engineering, many challenges and opportunities remain at the frontiers of the neural interface in terms of both neurobiology and engineering. In this short-communication, we explore critical needs in the neural engineering field to overcome these challenges. Disentangling the complexities involved in the chronic neural interface problem requires simultaneous proficiency in multiple scientific and engineering disciplines. The critical component of advancing neural interface knowledge is to prepare the next wave of investigators who have simultaneous multi-disciplinary proficiencies with a diverse set of perspectives necessary to solve the chronic neural interface challenge.

## 1. Introduction

Neurotechnologies that are capable of stimulating or recording from a small population of neurons have revolutionized quality of life by enabling the deaf to hear [1,2], the blind to see [3,4], and the paralyzed to write, grasp, and walk [5,6,7,8,9,10,11]. The advancement of this technology has seen a dramatic growth over the past decade which has attracted additional attention and increasing promises of what these devices can accomplish to further improve quality of life. These neurotechnologies can range from implants that are inserted deep within the nervous system to non-invasive wearable technologies that generally have more limited capabilities. Key progress feeding into the growth of this field is the investment from major pharmaceutical and start-up companies to provide alternatives to drugs with side-effects as well as increased congressional and government support in developing and maintaining the infrastructural apparatus for technology development. In parallel, advancements in batteries, wireless recharging, miniaturization, sensors, computer chips, and advancements in decoding algorithms and machine learning promise potential for dramatic advances in the coming decades. 

These neural interface technologies were originally employed as tools for basic science research in order to study how the brain works [12,13,14,15]. Basic science mapping experiments were carried out by using neural interfaces to electrically stimulate various regions of the brain or the nervous system and observing muscle twitches [16,17,18,19,20]. Mapping was also carried out in the opposite direction by applying sensory stimulation or driving motor activity and recording ionic currents from action potentials using microscale neural recording interfaces [21,22,23]. From these experiments, academic researchers discovered that specific functions of the nervous system were encoded in specific regions of the brain and nerve bundles [24,25,26,27,28,29]. Furthermore, they discovered that the frequency of action potentials recorded generally corresponded to the intensity of activity (sensation or muscle activation) [13,14,15,30,31]. These basic science discoveries have led to numerous neural interface applications from brain-computer interfaces that extract brain signals from paralyzed patients and allow them to control robotic limbs and computer cursors to electrical stimulation technologies that restore sensory function or treat Parkinson’s tremors [2,3,5,7,8,9,11,32,33]. The present short-communication takes a brief glance at the history of the field as well as a wide-angle perspective of the emerging challenges and opportunities on the horizon along the frontier of neural engineering.

## 2. Brief History of Microscale Implantable Neural Technologies

**Figure 1:**
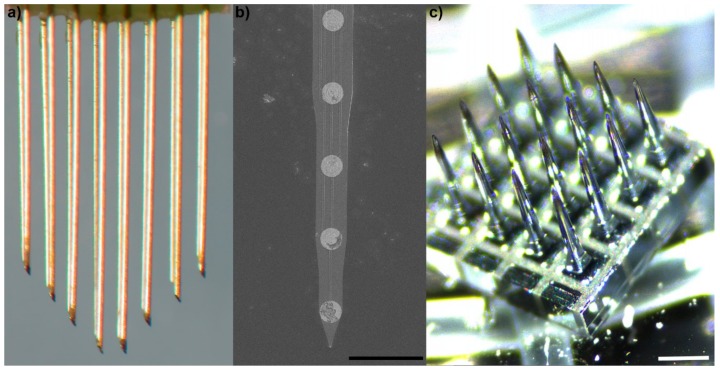
Classes of microscale implantable neural technologies: (**a**) 50 μm polyimide-insulated tungsten microwire with chiseled tips (Tucker–Davis Technologies, Alachua, FL, USA); (**b**) microfabricated silicon Michigan array with iridium electrode sites (NeuroNexus Technologies, Ann Arbor, MI, USA), scale = 100 μm; (**c**) macromachined boron-doped silicon array (Blackrock Microsystems, Salt Lake City, UT, USA), each needle is electrically separated at the base with glass. Scale = 400 μm.

**Figure 2:**
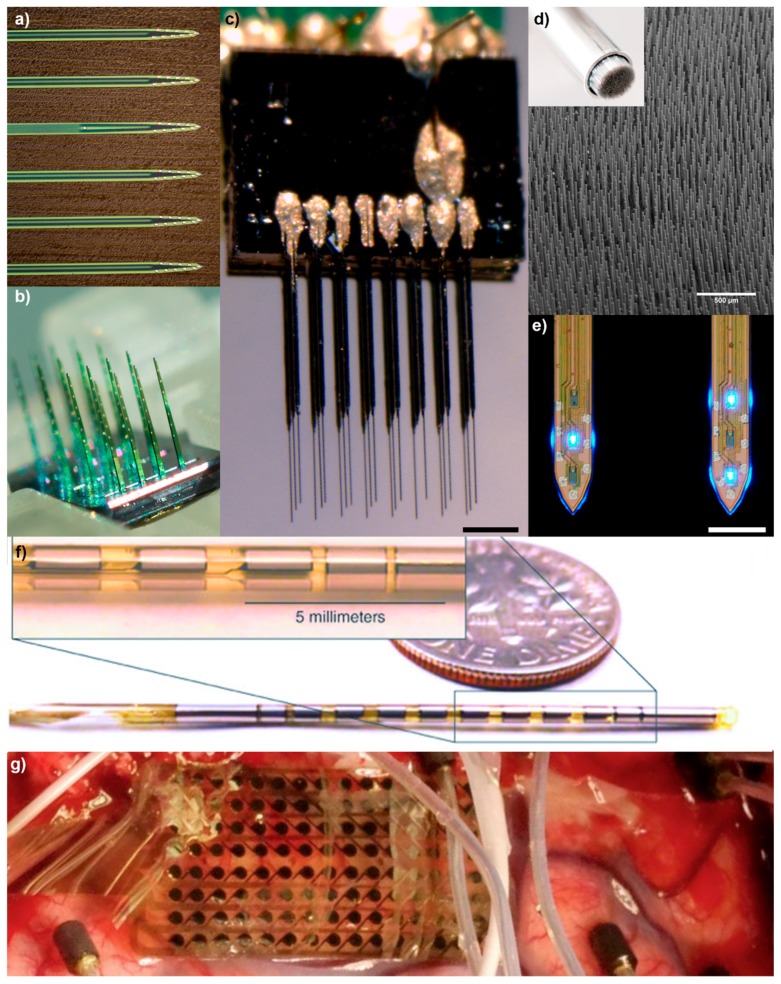
Advances in microscale neural interfaces: (**a**) 64-channel Buszaki Array (Neuronexus); (**b**) 128-channel Matrix Array (Neuronexus); (**c**) 24-channel ultra-small carbon fiber array on silicon stacks (courtesy of Paras Patel/Cynthia Chestek), scale = 100 μm; (**d**) high-density ultra-small microwire array (Paradromics Inc., San Jose, CA, USA), scale = 500 μm; (**e**) μLED silicon optoelectrode (courtesy of NeuroNex MINT Hub at University of Michigan, Ann Arbor, MI, USA (http://mint.engin.umich.edu)), scale = 100 μm; (**f**) a standard-sized 1.27 mm diameter Lawrence Livermore National Laboratories (LLNL) DBS-style penetrating probe constructed using microfabrication techniques, allowing for a higher-density of electrodes and avoiding typical hand-assembly techniques; and (**g**) A LNLL 128-channel microelectrocorticography (µECoG) array used for language mapping on awake patients. This 20-µm-thick flexible electrode array is constructed using thin-film polymers and metals and features 1.2 mm diameter electrodes.

Microscale neural interfaces were originally developed as research tools for academic investigation into the neural mechanisms that regulate attention, movement, and behavior [12]. Classically, these microscale interfaces have fallen into three categories: (1) microwire arrays (Figure 1a), (2) microfabricated planar arrays (Figure 1b), and (3) micromachined arrays (Figure 1c).

Microwire electrodes have two key components: (1) a conductive core wires, and (2) an insulator such as glass, parylene, teflon, or polyimide. Generally, the insulation is exposed at the recording site at the tip. Sometimes, other electrode site materials are deposited on the tip of the wire, before insulation or after removal of the insulation from the tip, in order to improve the electrical properties of the microelectrode. These wires are typically manually assembled into bed of needle arrays with several different strategies employed to align the wires [34]. 

Microfabricated planar arrays are typically engineered through photolithography of silicon, metals, and polymers [35]. These arrays are generally microfabricated through layering of multiple conductive and non-conductive materials leading to a planar configuration. While early planar arrays were made from rigid silicon (such as the Michigan arrays), flexible configurations have been developed, including planar arrays that can be rolled, folded, or stacked into 3D configurations [36].

Micromachined arrays are similar to microwire arrays. Instead of assembling individual wires into an array, a block of silicon is micromachined into a pillar of needles [37]. Band-saws are used to mill large blocks of conductive (boron-doped) silicon into individual pillars. During the milling process, non-conductive glass is used to hold the pillars in a bed of needle configuration. Once the square pillars are etched into round pillars, the electrode tip material and insulation are deposited onto the array in a manner similar to microwires. Due to the band-saw micromachining process, it is much more difficult to develop arrays that have staggered configurations when compared to microwire arrays. However, it is much easier to precisely align all of the needles to have the same angle.

These three array technologies form the basic classes of implantable microscale neural interfaces; however, the diversity within these classes has dramatically increased in both functionality and application (Figure 2). Advances in materials and biomaterials, microfabrication techniques, and packaging have enabled a large breadth of distinct configurations over a wide range of design space parameters [36,38,39,40,41,42,43,44,45,46,47,48,49,50,51,52,53,54,55,56,57]. Still, it is crucial to recognize that optimizing one key parameter often leads to trade-offs on other critical parameters, and failure to maintain the functional domain in each of the crucial parameter spaces will lead to a non-functional device [36]. For example, while flexible polymer devices are hypothesized to reduce tissue inflammation and improve the electrode-tissue interface, the materials and designs behind these compliant devices typically result in more brittle implants, increased resistance and lower signal conductivity, higher impedance, greater shunt leakage, and enhancement of motion related electromagnetic artifacts [36,58]. A comprehensive examination of technical advances and trade-offs in microscale technology design space parameters has been covered in a separate review [36]. While much of the technological development of neural interfaces has focused on improving electrically stimulating and recording from central nervous system (CNS) targets, some recent advances have fundamentally altered the traditional limits of neural implants. 

For example, optogenetics has dramatically altered the functionality of what once were exclusively electrical neural interfaces. Optogenetics includes transgenically expressing photon-gated ion channels called opsins in neuronal and non-neuronal cells whose cell activity are dependent on ion concentration [59]. Today, optogenetics also includes transgenically expressing fluorescent indicators into cells where the intensity level of the indicator changes based on the activity of the cells [60]. This is typically carried out by creating chimera proteins with a fluorescent protein, such as green fluorescent protein. The chimera is created such that the fluorescent protein is slightly denatured at rest. The other half of the chimera protein is designed to bind to key molecules of interest, such as calcium (released during action potentials) or glutamate [61,62,63]. The binding of the effector molecule leads to a conformation change in the binding site in the chimera, which rearranges the fluorescent protein into a conformation that allows the protein to fluoresce brightly, compared to the denatured state at rest. The adoption of optogenetic technology in the neuroscience community has motivated incorporation of waveguides as well as light-emitting and sensing diodes into microscale neural interfaces [36,40].

Similarly, a better understanding of the nervous system and foreign body response in the central nervous system has motivated the development of peripheral nerve interfaces. The central nervous system (CNS) is separated from the rest of the body by the blood-brain barrier (BBB). It was once believed that the brain was “immune privileged”. Today, this is understood to be an inaccurate dogma [64,65]. However, the inflammatory response and immune response that are triggered during surgical implantation of brain neural interfaces, as well as the threat of serious consequences from brain tissue infection along percutaneous connectors, have led investigators to search for less invasive neural interface approaches [66,67]. In parallel, new discoveries about the autonomic nervous system have led to the validation that modulating activity of peripheral nerves that feed into the brain can cause systemic physiological changes [24,25]. While early proof-of-concept studies utilized brain neural interfaces or modified brain neural interface technologies, interfacing with peripheral nerves requires dramatic differences in structure and design criteria compared to brain neural interfaces that are more suited to recording signals from neuronal cell bodies rather than axons.

Advancements in genetic engineering, biophysics, and a better understanding of functional connectivity and anatomy has opened up novel modalities for interfacing with the nervous system. In addition, as basic science understanding of the nervous system increases, it becomes possible to identify new targets for interfacing with the body and different aspects of physiology. Each new nervous system target requires a custom design in order to optimally interface with the nerve or neuron. This is especially true when interfacing the same peripheral physiological target across different animal models or different ages of the same model. Furthermore, it may be necessary, depending on the target, to consider “personalized device designs” similar to personalized medicine which accounts for person-to-person variability in clinical applications.

## 3. Challenges on the Horizon

Despite these numerous success stories, many challenges, and as a result, great opportunities remain unexplored [36,58,68]. There remains large variability in performance even between identical devices [69] due to both biological [58,67,70,71,72] and material integrity variance [73,74,75], even within the same subject [71,72]. Nevertheless, the field of neural engineering has reached a tipping point due to pioneers in neuroscience, technology development, and neurosurgery. Although many of the foundational components are primed for commercial growth of neural interfaces, there are still constraints in neural technology translation due to the unpredictability of discovery science. In addition, it remains highly risky to build a business plan around basic science breakthroughs. Therefore, big pharmaceutical companies have only recently started to gain confidence in foundational neural engineering science in order to invest in neurotechnology development. It is important to recognize that the considerable work necessary to advance the frontiers of neural interface science and lay the foundation for neural engineering had to come from tax-payers, government organizations (e.g., United States Department of Veterans Affairs (VA), Department of Defense (DoD), National Institute of Health (NIH), National Science Foundation (NSF)), and donors, rather than businesses. This foundational academic research is an educational and cultural process that is necessary but difficult to evaluate in terms of technology development due to the long time-scales between basic science discovery and developing technology applications [76]. However, because of the long time-scales, it is crucial to advocate for investing today, especially in order to avoid losing the tremendous academic, government, and industry momentum that has built up in the neural engineering field.

## 4. Need for the Science of Neural Engineering

Neural engineering is at crucial point, in which, unlike other established engineering industries, the basic scientific knowledge foundational for neural engineering is disproportionately incomplete. This limited understanding of the human brain shrouds undiscovered opportunities for advancement in neurotechnology. Biology is perhaps the most complex regulatory system known, and within biology, the nervous system is perhaps the most sophisticated control system that exists. As such, it is not possible to overpower biology with rudimentary physics and engineering. Instead, development of microscale neural interfaces requires a more challenging titration of increasing information bandwidth while minimizing injury and inflammation of the host tissue. Therefore, it is critical to continue advancing both technology development and neurobiology in parallel. 

Currently, the advancement of neuroscience is limited by the current capabilities of neurotechnology tools. Similarly, the development of devices is limited by the inadequate understanding of which designs and parameter trade-offs need to be optimized in order to maximize the extraction of meaningful neural signals [36]. Due to the long-time scales between basic science research and development of technology applications, the neural engineering field has long experienced deep criticisms on the shortage of clinical applications and aiding patients. Today, greater emphasis in neural engineering is placed on clinical impact over basic science research. However, in order to dramatically advance neural engineering, it is necessary to advance the science of neural engineering. In other words, it is necessary to continue to invest in the development of technology and studies that are designed to expand scientific knowledge rather than for therapeutic applications [22,46,58,74,77,78,79,80,81,82,83,84,85,86,87,88,89,90,91,92,93,94,95,96], even when the market segment is currently too small to support commercialization. 

For example, the standard Blackrock arrays have 400 μm shank pitch [97]. This is not because 400 μm is the optimal pitch to maximize signal detection or the optimal pitch to record from neighboring cortical columns. Studies in the hippocampus CA1 of rats showed that acutely the maximum recording radius of an extracellular electrode was 80–160 μm [98]. The 400 μm pitch was chosen because it was the width of the band-saw available at the time [37]. To this day, despite technological advancements that enable greater ranges of pitches, the physiologically optimal pitch for electrodes remains unknown. A major challenge for elucidating this optimal pitch is that it is necessary to evaluate a battery of different pitches individually. One might expect that a single design with a small pitch could easily allow oversampling to identify the optimal pitch for minimizing overlap. This would in theory identify the minimum pitch for enabling the densest recording configuration. However, the act of implanting the denser array leads to greater tissue strain, tissue response, and neurodegeneration, which ultimately alters the pattern of functional neurons around the implant [36,94]. 

The level of tissue response is also not limited to pitch, but also depends on the footprint of the probe, shape of tine, and surface chemistry of the interface, making it difficult to translate findings from one design to another [96,99]. Furthermore, there is an additional layer of complexity that is added due to the fact that the tissue response is dynamic and as a result, the optimal pitch is expected to also be dynamic over time [100,101]. The basic science discovery of identifying the optimal pitch has long-range impact on technology development. However, brain injury, neurodegeneration, neural regeneration, limited translatability across device designs, and immediate clinical impact and innovation is deemed to be too limited for current peer-review processes and commercial research.

Similarly, a major focus of research surrounding implantable neural interfaces are on neurons, implantable devices, and scar tissue around implants. However, rapidly growing evidence point to vasculature and glia as important regulators of neuronal health, network activity, and brain health [66,67,102,103,104,105]. Unfortunately, basic science studies aimed at understanding how glia and vascular dysfunction contribute to neural interface failure remain as long-range investments for improving neural interfaces and do not have immediate commercial value. These are only a few examples of many important topics that are critical to the overall advancement of the field, such as packaging (hermetic sealing) and glial-vascular interface technologies (as opposed to neural interface technologies) [36,68].

## 5. Need for Scientific and Engineering Convergence

Neural interface engineering requires a confluence of basic science, applied science, and engineering. For example, each anatomical target in the brain has distinct structures and circuit organization. Different brain regions are also composed of different structures of vascular network and different glial cell types as well as different ratios of neurons to glial cells. Even within neuronal cell-types, different regions of the nervous system are composed of uniquely diverse combination of excitatory and inhibitory neurons. This means that answering specific basic neuroscience questions can require technology designed for a specific target brain region and optimized to answer the specific question at hand. In other words, long-standing unanswered scientific questions could be better addressed by custom designs instead of a one-size-fits-all design. Unfortunately, from a financial point of view, a design that can only be applied to one specific experimental paradigm has limited commercial value due to a small and restricted market segment. Therefore, it is necessary to support academic infrastructures to accommodate technology development specifically designed to answer basic science questions.

The first steps to achieving this goal is that the engineers need to understand the anatomy, physiology, unintended consequences or “side-effects” of their designs, and the scientific principle behind the question their technology intended to answer. Similarly, scientists need to understand the limitations of materials, microfabrication techniques, failure modalities “in the field”, and design-driven technology development. Scientists need to guide technology development to optimally answer scientific questions without adding confounding variables to their study. Because functional microscale neural interfaces require fine titration of design parameters that are interdependent on each other [36], it is necessary for scientists to understand how achieving one optimal parameter can break functionality of other interdependent parameters. Therefore, engineering scientists and scientific engineers are both necessary in advancing the frontiers of the nervous system and integrating the newly found discoveries into technologies that interface directly or indirectly with the nervous system. For clinical applications, additional specialists are necessary including clinicians, patients, and caregivers or other “end-users” that interact with individuals who receive the neurotechnology.

The development of neurotechnologies requires a convergence of multiple disciplinary backgrounds including electrical engineering, electrochemistry, mechanical engineering, computer science, physics, biochemistry, biomechanics, material science, optics, biomaterials, packaging, ergonomics, molecular and cellular neurobiology, clinical science, and health care services. This requires both a wide breadth of expertise as well as enough cross-training depth to be able to integrate multiple engineering and scientific fields as well as end-user needs. While it is necessary to draw on multiple disciplines in the form of teams, the delays of the feedback loop between team members are limited by the speed in which team-members can communicate with each other. A commonly sought strategy to shorten that loop is to house multiple expertise in a single mind. However, this requires considerable cross-training time and effort on behalf of the individual. Given the growing scientific knowledge and accelerated advancement of engineering, it is becoming increasingly demanding for an individual to be fully proficient in all relevant scientific, engineering, and clinical expertise. Therefore, it is crucial for neural engineers to form teams of engineers, scientists, clinicians, and end-users as well as develop efficient communication techniques to reach the next level of technology development. While an increasing number of labs and programs strive to achieve this integration of science and engineering, this requires substantial contribution from individuals to learn, incorporate, and pass on training.

In turn, this means that the critical challenge for neural interface education and training is in converging neurobiology and neural engineering. Biology and engineering are often taught divergently with minimal overlap instead of being taught in an integrated and convergent manner. The nervous system is one of the most sophisticated computational systems, whose neural network activity is tightly regulated by the neural vascular unit and glia. Therefore, it stands to reason, as engineers, that by understanding the mechansims of how neurons, glia, and the neurovascular units regulate the neural network, it will be possible to identify new targets and means for interfacing with the nervous system in order to treat and repair diseases and injuries. However, in order to achieve this, it is necessary to bring together a diverse set of expertise, perspectives, and problem solving approaches, but have the capability to rapidly communicate with a common set of neuroscience and neural engineering “language”. 

## 6. Need for Diversity

While the ultimate goal of the BRAIN Initiative (NSF, NIH, etc.) and commercial Bioelectronic Medicine (Galvani Bioelectronics (GSK and Verily), NeuraLink, Kernel, etc.) is to understand brain function and treat neurological and physiological disease via the nervous system, the critical hurdle is placed on unreliable neuroelectronic interfaces over relevant time scales and the limited understanding in the neural interfacing field [36,67,68,97,106,107,108,109]. Just as a diversity of expertise is necessary to develop the next-generation microscale neural interfaces, it is necessary to have a diversity of perspectives and problem-solving approaches. The consequence of lack of diversity translates into limited diversity of opinion and perspectives, the blind spread of popular dogma, and the quenching of minority views. For example, a prevailing hypothesis in the field is that flexible devices will out preform traditional stiff implants. While plenty of evidence suggests that tissue injury is reduced around softer biomaterials, 50 years of polymer microelectrode research and limited success support the unpopular view that flexible polymer implants suffer from higher electrical impedance, higher resistivity, lower material strengths, higher shunt capacitance, larger device sizes, and new delamination issues that result in poorer performance compared to traditional devices [36,58]. This demonstrates issues in diversity as emphasized by NSF, “*Diversity—of thought, perspective, and experience—is essential to achieving excellence in 21st century science and engineering research and education*” [110]. Multi-disciplinary training in science and engineering are necessary, as well as diversity in perspective to understand the underlying problem and diversity in the approach of solving the problem. It is crucial to recognize that diversity in perspective and approach often stem from diversity in cultural and socio-economic backgrounds.

This diversity in approach to understanding the underlying problem and approach to problem-solving are deeply entangled with cultural and social backgrounds [111]. One study showed that gender diversity is correlated to 41% higher productivity compared to all-female or all-male teams [112]. Another study found that companies were 15% more likely to gain financial returns for companies in the top quartile of gender diversity and 35% more likely for companies in the top quartile for racial/ethnic diversity [113]. These studies add to a growing body of research that demonstrates gender, cultural, and ethnic diversity improves productivity, medical research, and clinical outcomes [114,115,116,117,118,119,120,121,122,123,124,125,126,127,128,129,130,131,132,133,134,135,136,137,138,139,140,141,142,143,144,145,146,147,148,149,150,151,152,153,154,155,156,157,158,159,160,161,162,163,164,165,166,167,168,169,170]. While similar studies in neural interface engineering have not been carried out, evidence in other fields suggest a potential for growth in the field by addressing gender and ethnic diversity. In a multi-disciplinary field such as microscale neural interface engineering, it is important for teams to have a diverse multi-disciplinary portfolio of ideas, skills, interests, technical background, and cultural and social backgrounds [111].

Therefore, it is crucial to protect and nurture researchers and prospective-researchers of underrepresented minorities who have been the victims of biases. In a seminal study by Rosenthal and Fode [171], half of wild-type littermates were randomly labeled “smart rats” and researchers were asked to compare the performance of these “smart rats” he “discovered” against the other half of the litter. What he showed was that the “smart rats” significantly out-performed their littermate clones in maze-tasks. He further described the Experimenter Expectancy Effect in which the experimenter’s bias leads to unconscious behavioral cues that in turn influence the behavioral outcome of the subject. While the potential of these clones should be statistically identical, the “normal rat” group did not reach their potential due to the interactions with the experimenter. Therefore, in promoting diversity, it is crucial to recognize the metrics, which are measures of past performance, do not represent future potential, in individuals who grew up in environments of bias including women, non-binary gender minorities, and ethnic minorities [172]. This further extends to the fact that “equal opportunity” cannot equate “equal distribution” until such time that all implicit biases are eliminated [172]. Similarly, multiple studies have demonstrated that affirmative action admittees with lower incoming scores have a higher predisposition to success [173,174,175]. Therefore, it is necessary to provide for underrepresented minorities to counter the history of bias, facilitate reaching their full potential, and contribute to the diverse perspectives and problem-solving approaches necessary to address the multifaceted challenges surrounding neural interfaces.

## 7. Conclusions

Microscale neural interfaces have demonstrated great potential in basic neuroscience research and clinical neuroprosthetics. While these early results have generated enormous enthusiasm, limitations, and challenges in reliability and large performance variability remain. In other words, there is much more to be explored and discovered at the frontiers of microscale neural interfaces. Pioneers that are advancing these frontiers will be better positioned with cross-training in microfabrication/biomaterials engineering and neurobiology/neuroscience, as well as assembling teams with a diverse set of technical expertise as well as culture backgrounds. This is because fundamental basic science research is an academic and cultural process, and as greater cultural diversity is intermingled into this process, richer and deeper discoveries will be generated.
